# Two seriously ill neonates born to mothers with COVID-19 pneumonia- a case report

**DOI:** 10.1186/s13052-020-00897-2

**Published:** 2020-09-21

**Authors:** Setareh Sagheb, Ameneh Lamsehchi, Mohamadreza Jafary, Reza Atef-Yekta, Kourosh Sadeghi

**Affiliations:** 1 Department of Neonatology, Shariati Hospital, Tehran University of Medical Sciences, Tehran, Iran; 2 Shariati Hospital, Tehran University of Medical Sciences, Tehran, Iran; 3 Department of Anesthesiology, Shariati Hospital, Tehran University of Medical Sciences, Tehran, Iran; 4 Department of Clinical Pharmacy, Tehran University of Medical Sciences, Tehran, Iran

**Keywords:** Neonate, Vertical transmission, COVID-19, SARS-COV-2, Case report

## Abstract

**Background:**

Coronavirus disease 2019 (COVID-19), a highly contagious viral disease has spread from Wuhan, Hubei Province, China to all over the world from its first recognition on December 2019. To date, only a few neonatal early-onset sepsis by SARS-COV-2 has been reported worldwide.

**Case presentation:**

In this report, we present two seriously ill neonates who were born from mothers with stablished COVID-19 pneumonia. Laboratory tests showed lymphopenia with high LDH and hypocalcemia right after the birth. They had fever for days without responding to antibiotics and despite ruling out other potential causes. Both patients had positive RTPCR for SARS-COV-2 in the second round of testing but the first assay tested was negative. Hydroxychloroquine was used to treat both patients; the first patient was treated with it over a period of 14 days before showing signs of improvement. The second patient responded to the treatment over a period of 5 days.

**Conclusion:**

Although based on the available evidences, vertical transmission of COVID-19 is less likely, many aspects of pathogenesis and transmission of this novel virus are still unclear. Therefore we cannot rule out the vertical transmission totally. Further investigations are warranted to determine the exact mechanisms and routes of transmission.

## Background

Since December 2019, the coronavirus disease (COVID-19), which is a highly contagious virus, has been rapidly spreading worldwide. Accordingly, it was firstly appeared in Wuhan, Hubei Province, China [1]. Coronavirus is an RNA virus, which can cause a wide spectrum of clinical manifestations in human from a common cold to severe acute respiratory syndrome (SARS) known as the SARS-associated coronavirus [2]. It was reported that Severe Acute Respiratory Syndrome coronavirus 2 (SARS-COV-2) similar to SARS-COV and Middle East Respiratory Syndrome Coronavirus (MERS-COV), does not have vertical transmission from mother diagnosed with COVID-19 infection to her fetus [3, 4]. However, reports of 3 neonates with early-onset COVID-19 infection in China pointed out that not much is understood on intrauterine transmission, so further investigations are required. To date, few neonates infected by COVID-19 have been reported [5]. In the following sections, we presented two neonates with COVID-19 positive reverse transcription polymerase chain reaction (RT-PCR) assay in Iran. These two affected neonates’ parents provided us with a written consent to report on their neonates. This survey was approved by the Medical Ethics Committee of Tehran University of Medical Sciences (IR.TUMS.VCR.REC 1399.46).

## Case presentation

### Case 1

A preterm (gestational age = 31 weeks) infant boy was born on March 3, 2020, in Shariati hospital, Tehran-Iran. His Apgar scores were 7 and 8 in 1 and 5 min after birth, respectively. Moreover, the weight at the time of birth was 1550 g. His 36-year-old mother (gravid: 2, abortion: 1) had shortness of breath and fever since about 7 days before delivery. In addition, she had positive RT-PCR test result for SARS-COV-2. Unfortunately, she died by passing 3 days from cesarean delivery. The baby had tachypnea and retraction after birth so he was transferred to NICU immediately, without performing skin to skin contact, with early initiation of continuous positive airway pressure (CPAP) therapy.

**Fig. 1:**
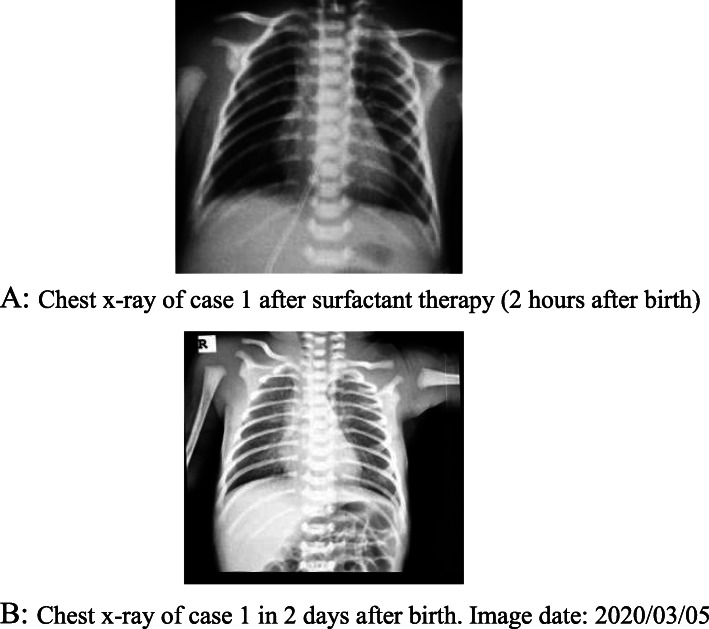
**a** Chest x-ray of case 1 after surfactant therapy (2 h after birth). **b** Chest x-ray of case 1 in 2 days after birth. Image date: 2020/03/05

At the first step, we washed the baby with lukewarm water to prevent probable transmission of COVID-19 from amniotic fluid to baby. Afterward, we isolated him in a separate room. Also, droplet and contact precautions were performed during transferring him to the NICU, and we also had strict precaution protocols for nurses who were taking care of him. Subsequently, he was intubated due to progressive severe respiratory distress. Then, he was injected by surfactant through endotracheal tube (ET) after intubation. Pharyngeal swab for SARA-COV-2 was sent to the laboratory within an hour after admission. Moreover, the first chest X-ray was performed after the injection of surfactant due to progressive respiratory distress. It showed a good aeration due to early intervention and ventilator support (Fig. 1a).

His first pharyngeal swab RT-PCR test for COVID-19 was negative. Two days after birth, his axillary temperature reached 38.5 °C (101.4 °F), and chest radiography performed for him, showed a diffuse opacification of both lungs with air bronchograms and hypoaeration (Fig. 1b).

Antibiotic regimen was upgraded and meningitis was also ruled out by performing lumbar puncture and negative cerebrospinal fluid (CSF) culture. Moreover, we checked serum & CSF herpes simplex viruses (HSV) PCR which were also negative. The results of nasopharyngeal multiplex PCR tests (for enterovirus, adenovirus, and H1N1) were also negative. A massive pulmonary hemorrhage complicated his situation on day 4 after his birth. In such case, we decided to treat him by 5 mg/kg/day of hydroxychloroquine that was administered via feeding tube, considering the probable COVID-19 infection. Due to the persistent high fever despite administrating antibiotics for 5 days (excluding (Hemophagocytic Lymphohistiocytosis (HLA)), viral and bacterial sepsis as explained earlier), we conducted a pharyngeal swab test again for COVID-19 RT-PCR assay on day 7 after his birth time, and the RT-PCR test result for COVID-19 was positive at this time (Table 1). As he was still suffering from fever (39 °C Axillary), we continued the administration of hydroxychloroquine until the fever stopped on day 16 after his birth (i.e. treatment lasted for a 14-day period). Hydroxychloroquine was well-tolerated by the patient without any significant clinical or laboratory adverse event. On his first laboratory tests, the baby had mild metabolic acidosis (PH: 7.24, PCO2:41 mmHg, PO2:60 mmHg, HCO3:17.6 mEq/L, BE:− 9.4 mEq/L), lymphopenia (2.4 × 10^9^ /L), the elevated LDH (1645 U/L normal: ≤450 U/L), and normal CRP (6 mg/dl). The results of renal and liver functions’ tests were within a normal range. Hyponatremia (124 mEq/L) with a high amount of urine sodium (Na: 128 mEq/L) and normal urine specific gravity (uSG: 1008) demonstrated the presence of syndrome of inappropriate antidiuretic hormone secretion (SIADH) on day 2 of his life. Moreover, he also had hypocalcemia (6.6 mg/dl) (Table 1). His normal brain sonography changed to grade ***Ш*,** IVH (Intraventricular Hemorrhage) through the first week. During hospitalization, both blood and urine culture tests’ results were negative. He was diagnosed with prolonged pulmonary hemorrhage lasted for 14 days; however, his massive pulmonary hemorrhage has well recovered. Furthermore, because of his mother death, we was not breastfed. Therefore, he was fed with formula, and during hospitalization, only his father could come to visit him with respect to all precautions and having at least 1.5 m distance from him. He was discharged in May with negative COVID-19 RT-PCR test’s results that was performed twice. In follow up, he has a cerebroventricular shunt because of hydrocephalous.

**Table 1:** Laboratory Findings of Case 1

Time	March 3			March 10
	Laboratory Test	Result	Normal Range	
	White blood cell count, 10^9^ /L	8.9	3–19	
	Lymphocyte count, 10^9^ /L	2.4	2.5–6	
	Lymphocyte ratio, %	27	≥30	
	Calcium, mg/dl	6.6	8.6-10.6	
	Lactat dehydrogenase, U/L	1645	≤450	
	PCR of nasopharyngeal swab	Negative	_	positive
	PH	7.24	7.35–7.45	
	HCO3, mEq/L	17.6	22–26	
	BE, mEq/L	-9.4	-2 - + 2	
	Pco2, mmHg	41	35–45	

### Case 2

**Fig. 2:**
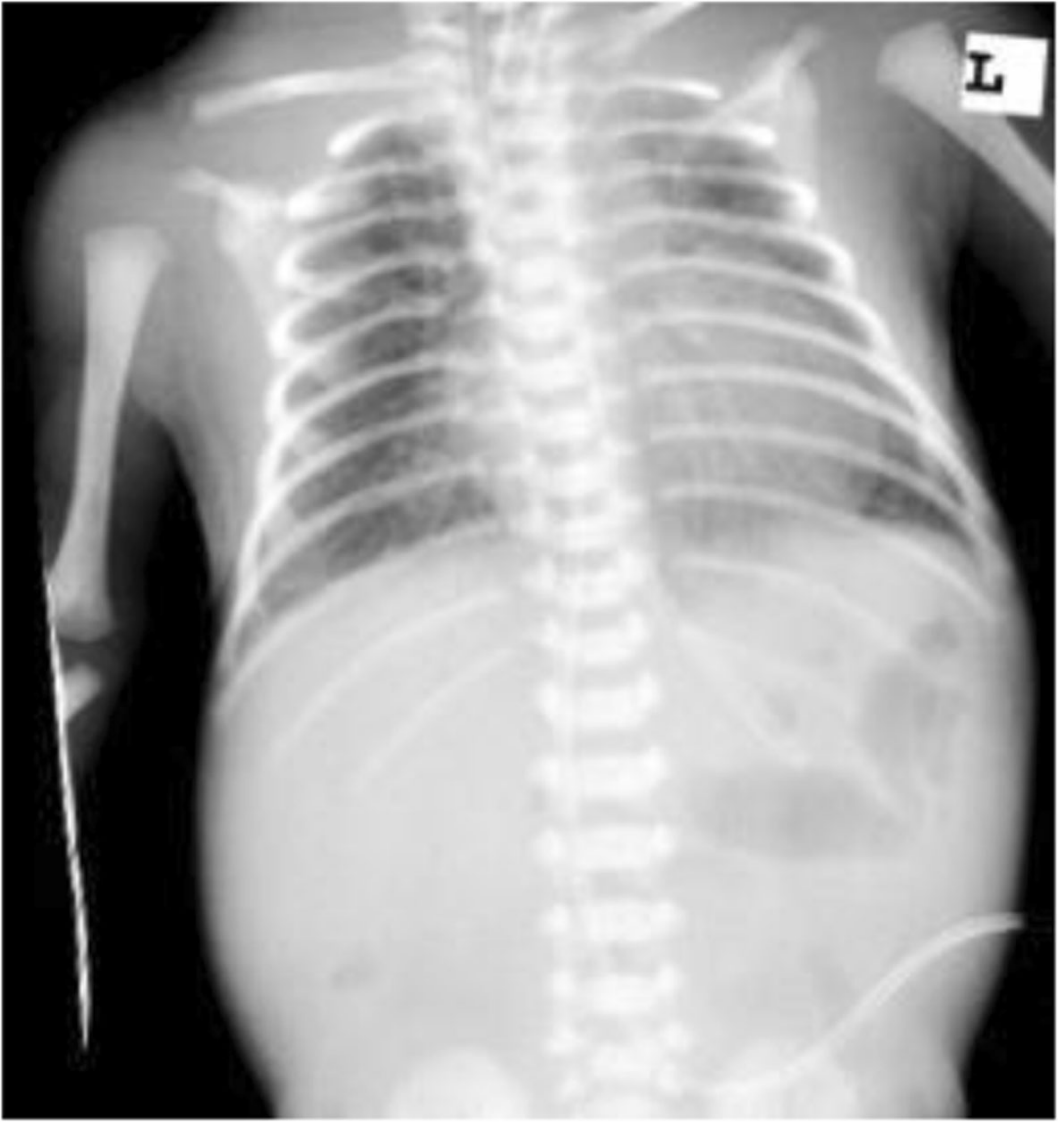
Chest x-ray of case 2. Image date: 2020/03/05

On March 5, 2020, a pregnant woman had emergency cesarean, because of her respiratory distress, and then she gave birth to a baby boy. She was suspected to having COVID-19 infection as she had fever and tachycardia, and also because she came from the city of Qom where the outbreak was started from in Iran. She had one other child (gravid: 2, living child: 1, abortion 0). Lung HRCT was performed for her and the CT scan pattern was similar to those found in SARS-COV − 2; however, her COVID-19 RT-PCR test was negative. The baby’s gestational age was 33 week + 4 days. Moreover, his Apgar scores were 7 and 8 in 1 and 5 min after birth respectively. Also, his weight at the time of birth was 1930 g. The baby had tachypnea and retraction soon after birth, without performing skin to skin contact; therefore he was transferred to NICU with early CPAP and then admitted in an isolated room. Similar to case 1, we performed all the instructions for droplet and contact precautions during transferring him to the NICU, and the standard nursing protocols for droplet and contact transmission were controlled during hospitalization. Subsequently, he was intubated due to progressive severe respiratory distress, so he received surfactant through ET. Surprisingly, the baby had fever at birth time, immediately when he was delivered. Two days later, his fever stopped and we could extubate him. By performing Chest X-ray, his lung showed a good aeration after receiving surfactant (Fig. 2).

His O2 saturation was 98% without oxygen therapy. The result of the first COVID-19 RT-PCR assay (within 1 h of birth) was negative. Notably, one nurse fed him with milk, which was prepared with an electric pump from his mother. Precautions for milking process were done, including washing breast, hands, and electric pump. The baby displayed no other symptoms until 12 days after his birth. By passing 12 days from birth, he had apnea and fever, so we performed sepsis workup while trying to exclude other causes of apnea. In this regard, the patient did not need oxygen support. While investigating the potential causes such as performing lumbar puncture, changing antibiotics, and giving caffeine, a second RT-PCR nasopharyngeal specimen for COVID-19 test was taken for testing purposes. The result of test performed on day 12 was positive for COVID-19. Therefore he was subsequently treated by hydroxychloroquine 5 mg/kg/day via a feeding tube for 5 days, hence after which the fever stopped. Also, chest X-ray displayed no particular pattern during the fever period. Similar to case 1, hydroxychloroquine was well-tolerated by the infant with no significant clinical or laboratory adverse event.

In his first blood tests, arterial blood gas analysis showed PH: 7.31, PCO2:34 mmHg, PO2:178 mmHg, HCO3:17.1 mEq/L, and BE:-8.2 mEq/L. Moreover he had lymphopenia (1.9× 10^9^ /L), the elevated LDH (963 U/L normal: ≤450 U/L), and low CRP (1.9 mg/dl). He also had hypocalcemia 7.7 mg/dl and CSF, blood, and urine culture tests were negative during admission (Table 2).

**Table 2:** Laboratory Findings of Case 2

Time		March 5		March 17
	Laboratory Test	Result	Normal Range	
	White blood cell count, 10^9^ /L	7	3–19	
	Lymphocyte count, 10^9^ /L	1.9	2.4–6	
	Lymphocyte ratio, %	27	≥30	
	Calcium, mg/dl	7.7	8.6-10.6	
	Lactat dehydrogenase, U/L	963	≤450	
	PCR of nasopharyngeal swab	Negative	_	positive

We discharged him with two negative SARS-COV-2 RT-PCR test results, which were performed with 24 h space. To date, he has not shown any abnormal symptoms after his recovery. For this case, follow up neurological testing will also be done in future.

## Discussion and conclusions

Presently, there is no specific guideline for the management of COVID-19 infection in neonates. As a result, usually, expert opinions and protocols for adult infection treatment and control are followed [6]. In our experience, hydroxychloroquine was used for both patients; and for the first case, a treatment course of 14 days appeared to be helpful. According to the lack of reliable scientific research on early-onset infection of COVID-19 in neonates, it may be assumed that the symptoms in the patients were caused by prematurity or sepsis, rather than SARS-COV-2 infection. Nevertheless, it is clear that our data on early-onset infection of COVID-19 is incomplete from many aspects. Recently, two reports [3, 4] announced that intrauterine transmission of SARS-COV-2 is not probable. They evaluated cord blood, amniotic fluid and even breast milk samples of mothers diagnosed with COVID-19 pneumonia, but SARS-COV-2 tests were negative in all cases. Furthermore, Zhu et al. observed ten neonates born from mothers infected by COVID-19 and suggested that SARS-COV-2 may cause several harmful effects such as premature labor, respiratory distress, thrombocytopenia or abnormalities in liver function tests or maybe death on neonates however, all their neonate’s COVID-19 RT-PCRs were negative [1]. In this report, our neonates had lymphopenia, the elevated LDH, and hypocalcemia immediately after birth time. The first case developed sign and symptoms of SIADH on the second day after his birth with onset of running a high temperature. Furthermore he developed a high anion gap metabolic acidosis on the 3rd day. After which, he suffered from the prolonged pulmonary hemorrhage on the 4th day of his birth. Normal Apgar scores of both cases in the absence of severe metabolic acidosis after birth, beside normal neurologic examination for gestational age ruled out asphyxia. Consequently, because of performing all the aforementioned droplet and contact precautions during hospitalization, having high LDH, lymphopenia and SIADH soon after birth may be due to early-onset infection of SARS-COV-2. One study in China reported that, 3 neonates born from 33 patients infected by SARS-COV-2 had an early-onset infection [5]. In our study, Chest X-Ray patterns were unremarkable in term of COVID-19 infection; however, the patients were too unstable to go through a CT scan during admission. Furthermore, another study conducted on a limited number of patients showed a high level of SARS-COV-2 IgM in neonates born from COVID-19 infected mothers within 2 first hours of their birth [7], which may indicate infection transmission from mother to fetus. Also, we believe that we had strict operational procedures ensuring the prevention of infection transmission during labor and after transferring neonates to the isolated ward. However, since the birthdays are close to each other (March 3 and March 5 in 2020), a common denominator for infection can be possible in this short timeframe, and horizontal transmission in these cases cannot be ruled out. It is worth noting that, although our neonates’ RT-PCR tests’ results for SARS-COV-2 were negative 1 hour after their birth, they tested positive on day 7 and 12. Furthermore, Zou et al. in China [8] surveyed the relationship between viral loads of SARS-COV-2 in RT-PCR test, and the timing of swab sampling after symptoms were also detected. It seems that sensitivity of RT-PCR test depends on the source of sampling (e.g. throat, nose, etc.), time of sampling and the health care provider’s skill for performing an accurate sampling.

To the best of our knowledge, only a few neonatal early-onset sepsis by SARS-COV-2 have been reported worldwide. Despite older children usually show mild symptoms related to COVID-19 infection, neonates with COVID-19 infection typically exhibit an expanding range of clinical features. Moreover, it seems that, preterm neonates may have more severe symptoms compared to term neonates in this infection, which can be attributed to a weak immune system of the preterm neonates. Considering limited evidence, the priority should be given to clinical features of neonates, especially fever; once overriding RT-PCR test results.

During the current coronavirus pandemic, we suggest considering the treatment of SARS-COV-2 infection in seriously ill neonates with persistent fever and history of COVID-19 pneumonia in their mothers. Obviously, other potential causes must also be considered and excluded beforehand. It can be concluded that determining the preventive strategies and early detection of COVID-19 in neonates along with implementation of standard protocols for treatment, require further in-depth investigations. Also, the exact possibility of vertical transmission of COVID-19 during pregnancy still needs to be determined.

## Data Availability

All data including patients’ medical records, images and laboratory data are kept in our hospital for the minimum of 5 years based on the local regulations.

## Abbreviations

ALT: Alanine aminotransferase
AST: Aspartate aminotransferase
BUN: Blood urea nitrogen
COVID-19: Coronavirus disease 2019
CPAP: Continuous positive airway pressure
CPC: Choroid plexus cyst
CRP: C-reactive protein
CSF: Cerebrospinal fluid
ET: Endotracheal tube
HLH: Hemophagocytic Lymphohistiocytosis
HSV: Herpes simplex viruses
IVH: Intraventricular Hemorrhage
LDH: Lactate dehydrogenase
MERS-COV: Middle East respiratory syndrome coronavirus
NICU: Neonatal intensive care unit
PFO: Patent foramen ovale
RDS: Respiratory Distress Syndrome
RT-PCR: Reverse transcription polymerase chain reaction
SARS-COV-2: Severe acute respiratory syndrome Coronavirus-2
SIADH: Syndrome of inappropriate antidiuretic hormone secretion
TPN: Total parenteral nutrition
WHO: World Health Organization
